# Lipidomic Profiling Reveals HSD17B13 Deficiency-Associated Dysregulated Hepatic Phospholipid Metabolism in Aged Mice

**DOI:** 10.3390/metabo15060353

**Published:** 2025-05-27

**Authors:** Cong Zhang, Yingxin Feng, Xiaoyan Zhang, Youfei Guan, Wen Su

**Affiliations:** 1 Advanced Institute for Medical Sciences, Dalian Medical University, Dalian 116044, China; 18742565327@163.com; 2 Department of Pathology, Shenzhen University, Shenzhen 518060, China; 3 Health Science Center, East China Normal University, Shanghai 200241, China; wserien@163.com; 4 Department of Physiology and Pathophysiology, School of Basic Medical Sciences, Dalian Medical University, Dalian 116044, China

**Keywords:** HSD17B13, aging, liver, lipidomic, phospholipids

## Abstract

Objectives: HSD17B13 (17β-hydroxysteroid dehydrogenase 13), a lipid droplet-associated enzyme, has emerged as a key regulator of hepatic lipid metabolism and a potential therapeutic target for metabolic-associated fatty liver disease (MAFLD). While its role in lipid homeostasis and liver inflammation has been partially revealed, the impact of HSD17B13 deficiency on lipid metabolism in aged mice remains poorly understood. In this study, we performed comprehensive lipidomic profiling of liver tissues from aged *Hsd17b13* gene knockout (*Hsd17b13* KO) mice to investigate the effects of *Hsd17b13* deletion on hepatic lipid composition and metabolic pathways. Methods: Changes in hepatic lipid profiles were assessed through a liquid chromatography-tandem mass spectrometry (LC-MS/MS)-based lipidomic analysis. Results: The lipid profiles, including triglycerides (TGs), diglycerides (DGs), phosphatidylcholines (PCs), phosphatidylethanolamines (PEs), phosphatidylglycerols (PGs), and ceramides (Cers), exhibited notable alterations in the *Hsd17b13* KO mice. Conclusions: HSD17B13 plays a pivotal role in liver lipid metabolism during aging, and it is involved in the regulation of hepatic phospholipid metabolism. Our study highlights the importance of HSD17B13 in maintaining liver lipid homeostasis and its potential as a therapeutic target for age-related liver diseases.

## 1. Introduction

Aging is a major global health challenge, contributing to approximately two-thirds of daily fatalities worldwide [1,2]. The prevalence of chronic diseases, such as ischemic heart disease, stroke, diabetes, liver and kidney disorders, neurodegenerative conditions, and various cancers, increases significantly with advancing age [3,4]. Among elderly individuals, liver disease has emerged as a leading cause of mortality, with its incidence rising globally [5,6]. Aging exacerbates liver pathology through multiple mechanisms, such as a diminished regenerative capacity, increased lipid accumulation, and the accelerated progression of fibrosis [7]. Furthermore, changes in the gut microbiota, metabolism, and the immune microenvironment due to aging contribute to the development and progression of liver diseases [1,5,8]. However, the molecular mechanisms underlying age-related liver diseases remain poorly understood, necessitating further investigation to identify potential therapeutic targets.

17β-Hydroxysteroid dehydrogenase 13 (HSD17B13) is a lipid droplet-associated enzyme predominantly expressed in the liver [9]; it belongs to the HSD17B family, which catalyzes the interconversion between 17-keto- and 17-hydroxysteroids [10]. The *Hsd17b13* gene was first isolated from a human liver cDNA library in 2007 and initially named short-chain dehydrogenase/reductase family member 9 [11]. HSD17B13 is specifically localized to the lipid droplets (LDs) of hepatocytes, and its expression is significantly elevated in the livers of patients with metabolic-associated fatty liver disease (MAFLD) and mice fed with a high-fat diet [12,13], suggesting a critical role in lipid metabolism and disease progression. Variants of the *HSD17B13* gene, such as rs72613567 and rs6834314, have been linked to a lower risk of chronic liver disease progression in patients with MAFLD, including advanced fibrosis and cirrhosis [13,14]. It has been previously reported that *Hsd17b13* variants provide protection against metabolic dysfunction-associated steatohepatitis (MASH)-induced hepatic fibrosis by modulating pyrimidine catabolism [15]. Additionally, the pharmacological inhibition of HSD17B13 has shown promise in alleviating hepatic steatosis, although its effect on fibrosis remains controversial [16]. We recently reported that the phosphorylation of the Ser33 of HSD17B13 by protein kinase A could ameliorate MASH [17].

Phospholipids, including phosphatidylcholines (PCs) and phosphatidylethanolamines (PEs), are essential components of cellular membranes, playing crucial roles in maintaining membrane integrity and fluidity, as well as signal transduction, hence contributing to proper cellular functioning [18]. Their biosynthesis, primarily occurring in the endoplasmic reticulum through the conserved Kennedy pathway, is intertwined with nucleotide metabolism and redox balance [19,20]. Dysfunctional phospholipid metabolism is involved in the pathogenesis of liver diseases. In lipidomic studies of plasma obtained from patients with HBV-related liver diseases, the biosynthesis of ether PCs (PC-O species) was found to be disrupted, which resulted in elevated PC-O and reduced PC levels, suggesting that these lipids may serve as early indicators of hepatocellular carcinoma [21]. Moreover, alterations in the metabolic pathways of PE or its relative abundance can impair liver function through multiple mechanisms [22]. Notably, the ratio of PC to PE levels (PC/PE) is decreased in MAFLD patients than healthy controls. This means that dysregulated PC metabolism and discordant enzymatic activity of PE-N-methyltransferase (PEMT) are hallmarks of MAFLD progression [23].

The role of HSD17B13 in age-related liver diseases remains understudied. Given the increasing prevalence of MAFLD in aging populations, elucidating the impact of HSD17B13 on hepatic lipid metabolism during aging is of critical importance. The study presented herein addresses this gap by investigating the lipidomic profiles of liver obtained from aged *Hsd17b13* gene knockout (KO) mice, aiming to provide new insights into the role of this enzyme in age-related liver disease. Moreover, the potential of the enzyme as a therapeutic target is also evaluated.

## 2. Materials and Methods

### 2.1. Animals

Global *Hsd17b13* KO mice were generated as previously described [24]. Ten wild-type (WT) and nine *Hsd17b13* KO male mice were maintained for 22 months under specific pathogen-free conditions in the animal facility of Shenzhen University Medical School (Shenzhen, Guangdong, China). All mice were housed in a controlled environment with a temperature of 22 ± 1 °C, a humidity of 65% ± 5%, and a 12-h light-dark cycle (8:00 AM–8:00 PM). In this study, we used standard concentrated feed for mice aged 3 weeks or older, provided by Xietong Pharmaceutical & Bio-Engineering Co., Ltd. (Nanjing, Jiangsu, China; Cat# 1010013). The feed primarily consisted of ground corn, wheat, fish meal, chicken meal, dehulled soybean meal, soybean oil, and sufficient amounts of amino acids, vitamins, and minerals. Standard rodent chow and sterile water were provided ad libitum throughout the study. All animal procedures and care were conducted in strict accordance with the ethical standards and protocols outlined by the National Institutes of Health (US) [25], and they were approved by the Animal Care and Use Committee of Shenzhen University Medical School (ethics number IACUC-202300023). After the experiments, all animals were euthanized humanely.

### 2.2. Liver Pathological Assessments

For the histological examination, liver tissues were first fixed in 4% neutral-buffered formalin, then embedded in paraffin, and subsequently thin-sliced into sections, which were stained using hematoxylin and eosin (H&E), strictly following the manufacturer’s protocols. For lipid staining, additional liver samples were fixed in formalin and embedded in optimal cutting temperature compound. To highlight lipid deposits, the fresh frozen sections were fixed in 4% paraformaldehyde, briefly rinsed with PBS, and then stained with freshly filtered Oil Red O working solution for 10 min at room temperature. After staining, the sections were rinsed briefly in 60% isopropanol for 5 min and counterstained with hematoxylin for 15 s to enhance the cellular details.

### 2.3. Determination of Liver Triglyceride (TG) and Cholesterol (TC) Levels

Next, 20 mg of liver tissue was placed into 1 mL of an extraction solvent system consisting of chloroform and methanol at a volume-to-volume ratio of 2:1 (*v*/*v*) and then homogenized. The chloroform/methanol 2:1 mixture was allowed to stand at 4 °C for 18 h. Subsequently, 300 μL of water was added, and the mixture was centrifuged at 12,000 rpm for 10 min to separate the phases. After centrifugation, the lower organic phase was carefully transferred to a new tube and dried using a vacuum centrifuge. The dried lipids were then reconstituted in 200 μL of 5% Triton X-100. Liver TG and TC concentrations were measured using specific assay kits from BIOSINO (Beijing, China). All procedures were strictly performed in accordance with the manufacturer’s instructions.

### 2.4. Glucose and Insulin Tolerance Tests

Before the glucose tolerance test (GTT), the mice were subjected to a 16 h fasting period and then intraperitoneally injected with glucose at a dosage of 2 g/kg body weight. For the insulin tolerance test (ITT), the mice were fasted for 6 h prior to an intraperitoneal injection of insulin at a dosage of 0.5 U/kg body weight. Blood glucose concentrations were measured from tail blood samples using a glucometer at time points of 0, 15, 30, 60, and 120 min after the injection.

### 2.5. Lipidomic Analysis

#### 2.5.1. Lipid Extraction

Next, 100 mg of liver tissue was placed in a 2 mL centrifuge tube, and 750 μL of mixed solvent (chloroform-methanol, 2:1, *v*/*v*) was added at −20 °C. The mixture was vortexed for 30 s. Two steel balls were added, and the sample was homogenized in a tissue grinder for 60 s at 50 Hz, which was repeated twice. The tube was then placed on ice for 40 min, followed by the addition of 190 μL of water and vortexed for 30 s. The sample was incubated on ice for an additional 10 min. The mixture was centrifuged at 12,000 rpm for 5 min at room temperature, and 300 μL of the organic layer was transferred to a new centrifuge tube. Another 500 μL of mixed solvent (chloroform-methanol, 2:1, *v*/*v*) was added, vortexed for 30 s, and centrifuged at 12,000 rpm for 5 min at room temperature. The organic layer (400 μL) was transferred to the same tube. The samples were concentrated to dryness under vacuum and dissolved in 200 μL of isopropanol. The supernatant was filtered through a 0.22 μm membrane to obtain the prepared samples for a liquid chromatography-tandem mass spectrometry (LC-MS/MS) analysis [26,27].

#### 2.5.2. LC-MS/MS-Based Lipidomic Analysis

##### Chromatography

Chromatographic separation was performed using an ACQUITY UPLC^®^ BEH C18 column (2.1 mm × 100 mm, 1.7 μm, Waters) maintained at 50 °C. The autosampler temperature was set to 8 °C. Gradient elution was achieved with mobile phases A2 (acetonitrile-water = 60:40, containing 0.1% formic acid and 10 mM ammonium formate) and B2 (isopropanol-acetonitrile = 90:10, containing 0.1% formic acid and 10 mM ammonium formate) at a flow rate of 0.25 mL/min. A 2 μL injection volume was used after equilibration. The gradient program was as follows: 0–5 min, 70%-57% A2; 5–5.1 min, 57%–50% A2; 5.1–14 min, 50%–30% A2; 14–14.1 min, 30% A2; 14.1–21 min, 30%–1% A2; 21–24 min, 1% A2; 24–24.1 min, 1%–70% A2; 24.1–28 min, 70% A2 [28]. LC-MS/MS-grade isopropyl alcohol, acetonitrile, and methanol were purchased from Fisher Scientific (Loughborough, UK). Chloroform was sourced from Sinopharm (Shanghai, China), formic acid was sourced from TCI (Shanghai, China), and ammonium formate was sourced from Sigma-Aldrich (Shanghai, China). Ultrapure water was generated using a Milli-Q system (Millipore, Bedford, MA, USA).

##### Mass Spectrometry

Electrospray ionization-multiple-stage mass spectrometry experiments were conducted with a spray voltage of 3.5 kV and 2.5 kV in the positive and negative modes, respectively. Sheath gas and auxiliary gas were set to 30 and 10 arbitrary units, respectively. The capillary temperature was 325 °C. The Orbitrap analyzer scanned over a mass range of *m*/*z* 150–2000 for a full scan at a mass resolution of 35,000. Data-dependent acquisition MS/MS experiments were performed with high-energy collisional dissociation scans. The normalized collision energy was 30 eV. Dynamic exclusion was implemented to remove some unnecessary information in the MS/MS spectra [29]. The liquid chromatography analysis was performed on a Vanquish Ultra-High Performance Liquid Chromatography System (Thermo Fisher Scientific, Waltham, MA, USA). Mass spectrometry was conducted on a Q Exactive instrument (Thermo Fisher Scientific, Waltham, MA, USA).

#### 2.5.3. Statistical Analysis

The raw data were first converted to a compatible format and then imported into LipidSearch version 4 for comprehensive preprocessing, which included data collection, alignment, and normalization. Lipid annotation was achieved by matching precursor ion m/z values and production patterns to the LipidSearch database.

To correct for batch effects, the data were normalized using the total peak area. Subsequently, log transformation and Pareto scaling were applied to prepare the data for a statistical analysis. Unpaired *t*-tests were used to compare the means between groups, and Benjamini-Hochberg correction was applied to the obtained *p*-values for false discovery rate (FDR) control, with significance defined as *p* < 0.05 and variable importance in projection (VIP) > 1.

Multivariate statistical analyses were performed using both unsupervised and supervised models, including partial least squares-discriminant analysis (PLS-DA). Lipids with a *p*-value < 0.05 and a VIP score > 1 were considered significant for group discrimination.

For an in-depth lipidomic analysis, the structural features of the differentially identified lipids were analyzed using the lipidomoR package (version 0.1.2). A functional enrichment analysis of lipids from distinct communities was carried out using the LION package (version 0.1.0) [30,31].

## 3. Results

### 3.1. Liver Morphology of the Aged Hsd17b13 KO Mice

**Figure 1:**
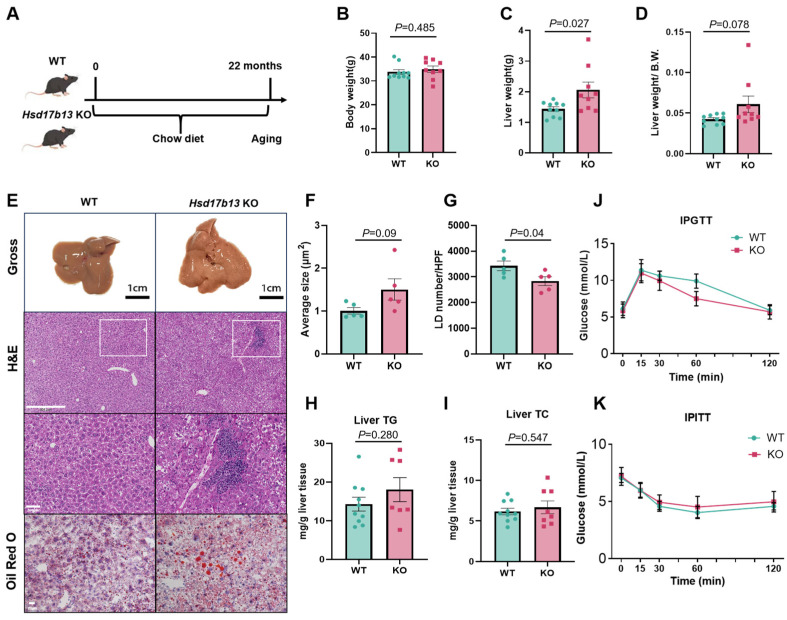
Liver morphology of aged *Hsd17b13* KO mice. (**A**) Experimental design: Analyses were performed in 22-month-old WT and *Hsd17b13* KO mice. Comparisons included body weight (**B**), liver weight (**C**), liver-to-body weight ratio (**D**), and gross liver morphology (scale bars = 1 cm) (**E**), histological assessment employed hematoxylin and eosin (H&E) (scale bars = 500 μm) and Oil Red O staining (scale bars = 20 μm), lipid droplet (LD) average size (**F**) and number (**G**) were quantified per high-power field (HPF). Hepatic total triglycerides (TGs) (**H**) and total cholesterol (TCs) (**I**) levels were measured. Intraperitoneal glucose tolerance test (IPGTT) (**J**) and insulin tolerance test (IPITT) (**K**) were conducted (WT: *n* = 10; KO: *n* = 9 biologically independent animals).

Marion et al. reported enlarged LDs in 9-month-old *Hsd17b13* KO mice [32]. To investigate the long-term consequences of HSD17B13 deficiency, we established an aged mouse model by maintaining wild-type (WT) and *Hsd17b13* KO mice on standard chow for 22 months (Figure 1A). Although there was no difference in their body weight (Figure 1B), the *Hsd17b13* KO mice had an increased liver weight (Figure 1C) and a trend toward an increased liver weight-to-body weight ratio (Figure 1D). We then performed a histopathological evaluation of the livers of the WT and *Hsd17b13* KO mice. In parallel with Marion’s observation, H&E staining revealed heightened inflammatory infiltration in the *Hsd17b13* KO mice (Figure 1E). Utilizing Oil Red O staining, we observed a notable trend toward an increased average size and a decreased number of LDs in the liver of the *Hsd17b13* KO mice (Figure 1F,G), which was not accompanied by a significantly increased hepatic deposition of neutral lipids (TGs and TCs) (Figure 1H,I). We also investigated glucose metabolism by conducting GTTs and ITTs on both groups of mice. There was no notable difference in the glucose tolerance and insulin sensitivity between the two studied groups (Figure 1J,K). Our data indicate that an HSD17B13 deficiency in mice is accompanied by the development of aging-related hepatitis, without affecting glucose metabolism.

### 3.2. Liver Lipidome Is Altered in the Aged Mice Deficient in the HSD17B13 Enzyme

**Figure 2:**
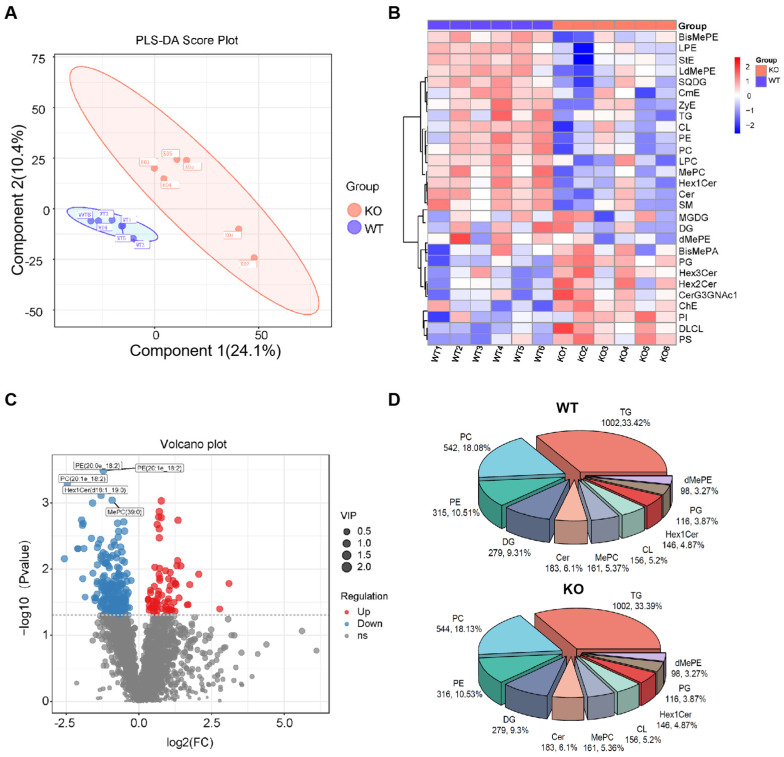
Lipidomic profiling reveals altered lipid metabolism in the *Hsd17b13* KO mice. (**A**) The partial least squares-discriminant analysis (PLS-DA) score plot presents the lipidomic data derived from the WT and *Hsd17b13* KO mice. (**B**) Heatmap showing the relative abundances of differently lipid subclasses between the WT and *Hsd17b13* KO mice. (**C**) Volcano plot visualizing the changes in the lipid profiles between the WT and *Hsd17b13* KO mice. (**D**) Pie charts representing the distribution of lipid categories in both the WT and *Hsd17b13* KO mice. Bis-methylphosphatidylethanolamine (BisMePE); lysophosphatidylethanolamine (LPE); stigmasteryl ester (StE); lysodimethylphosphatidylethanolamine (LdMePE); sulfoquinovosyldiacylglycerol (SQDG); carnitine ester (CmE); zymosterol ester (ZyE); triglyceride (TG); cardiolipin (CL); phosphatidylethanolamine (PE); phosphatidylcholine (PC); lyso-phosphatidylcholine (LPC); methylphosphatidylcholine (MePC); monohexosylceramide (Hex1Cer); ceramide (Cer); sphingomyelin (SM); monogalactosyldiacylglycerol (MGDG); diglyceride (DG); dimethylphosphatidylethanolamine (dMePE); bis-methylphosphatidic acid (BisMePA); phosphatidylglycerol (PG); trihexosylceramide (Hex3Cer); dihexosylceramide (Hex2Cer); ceramide trihexoside-N-acetylglucosamine (CerG3GNAc1); cholesteryl ester (ChE); phosphatidylinositol (PI); dilyso-cardiolipin (DLCL); phosphatidylserine (PS). (WT: *n* = 6; KO: *n* = 6 biologically independent animals.)

To investigate whether lipidome alterations accompanied the HSD17B13 deficiency-driven liver hepatitis in the aged mice, we conducted untargeted lipidomic profiling of the livers of the 22-month-old WT and *Hsd17b13* KO mice. Liver lipids were extracted using a chloroform/methanol protocol and analyzed using MS/MS in both positive and negative ionization modes. A multivariate analysis using partial least squares discriminate analysis (PLS-DA) demonstrated robust metabolic segregation between genotypes (R^2^Y[cum] = 0.991, Q^2^[cum] = 0.67), confirming minimal model overfitting (Figure 2A). The hierarchical clustering of differentially expressed lipid species demonstrated distinct lipidome signatures in two genotypes (Figure 2B). In the *Hsd17b13* KO mice, lysophosphatidylethanolamine (LPE), PE, PC, and cardiolipins (CLs) were downregulated, while phosphatidylglycerols (PGs), trihexosylceramide (Hex3Cer), dihexosylceramide (Hex2Cer), dihexosyl *N*-acetylhexosyl ceramide (CerG3GNac1), cholesterol ester (ChE), phosphatidylinositol (PI), and phosphatidylserine (PS) were upregulated. A volcano plot illustrating the differentially expressed lipid species between the WT and *Hsd17b13* KO groups is shown in Figure 2C. The five most notably downregulated lipid species were PC O-20:1_18:2, PE O-20:0_18:2, monohexosylceramide (Hex1Cer) d18:1/19:0, PE O-20:1_18:2, and methylphosphatidylcholine (MePC) 39:0. Global lipid class profiling revealed TGs as the predominant component (33.39% vs. 33.42%), followed by PCs (18.13% vs. 18.08%) and PEs (10.53% vs. 10.51%), across all groups (Figure 2D).

### 3.3. Altered TG and Diglyceride (DG) Metabolism Landscape in the Hsd17b13 KO Mice

**Figure 3:**
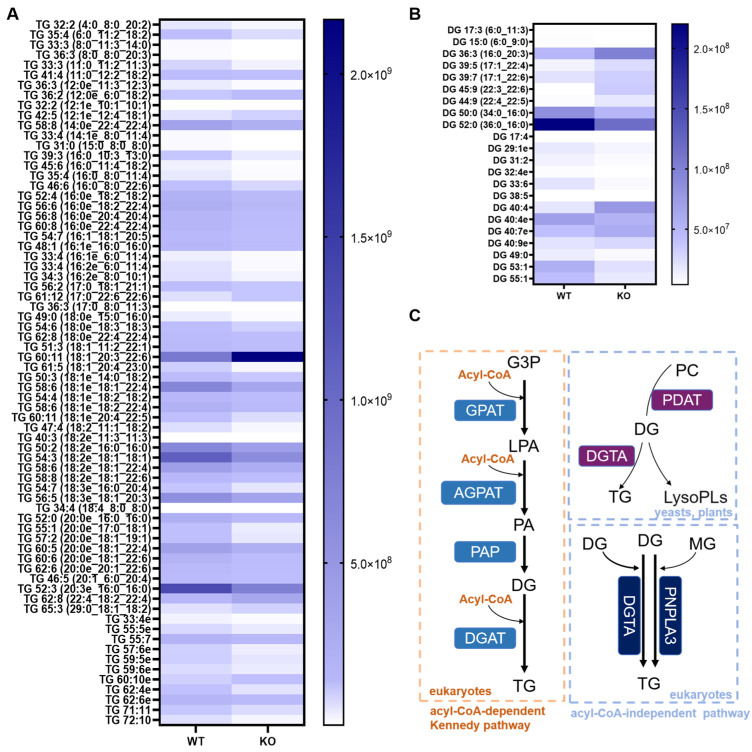
Altered TG and DG metabolism landscapes in the *Hsd17b13* KO mice. A heatmap illustrating the differences in (**A**) TG and (**B**) DG. Additionally, an illustrative diagram (**C**) depicts TG biosynthesis through the acyl-CoA-dependent Kennedy pathway (orange box) and the acyl-CoA-independent pathway (blue box). Glycerol-3-phosphate acyltransferase (GPAT); 1-acyl-glycerol-3-phosphate O--acyltransferase (AGPAT); phosphatidic acid phosphatase (PAP); diacylglycerol acyltransferase (DGAT); Phospholipid: diacylglycerol acyltransferase (PDAT); acyl-CoA-independent diacylglycerol transacylase (DGTA); patatin-like phospholipase domain-containing protein 3 (PNPLA3); glycerol-3-phosphate (G3P); lysophosphatidic acid (LPA); phosphatidic acid (PA); lysophospholipids (LysoPLs); monoacylglycerol (MG).

Considering the importance of triacylglycerols in lipid metabolism in hepatocytes, we carried out comprehensive omics sequencing targeting TGs; then, we performed a heatmap analysis on the obtained sequencing data. The heatmap analysis visually presented the data, showing that TG 18:1_20:3_22:6 was the most clearly upregulated (Figure 3A). The top three most upregulated DGs were DG 22:3_22:6, DG 40:4, and DG 22:4_22:5 (Figure 3B). In eukaryotes, the acyl-CoA-dependent Kennedy pathway involves three sequential acylation reactions of glycerol-3-phosphate. Through a series of acylation reactions, glycerol-3-phosphate is sequentially converted to lysophosphatidic acid, phosphatidic acid, and then diacylglycerol. Finally, DG is acylated with a third fatty acyl-CoA molecule by diacylglycerol acyltransferase (DGAT), forming TG, which can be stored for energy or used in other cellular processes. In addition, another TG synthesis pathway is the acyl-CoA-independent pathway, which is mediated by acyl-CoA-independent diacylglycerol transacylase (DGTA) and patatin-like phospholipase domain-containing protein 3 (PNPLA3) separately [33,34]. These enzymes transfer a fatty acyl moiety from a phospholipid (such as PC) to DG to form TG in yeasts and vascular plants (Figure 3C) [35].

### 3.4. Altered Phospholipid Metabolism in the Hsd17b13 KO Mice

**Figure 4:**
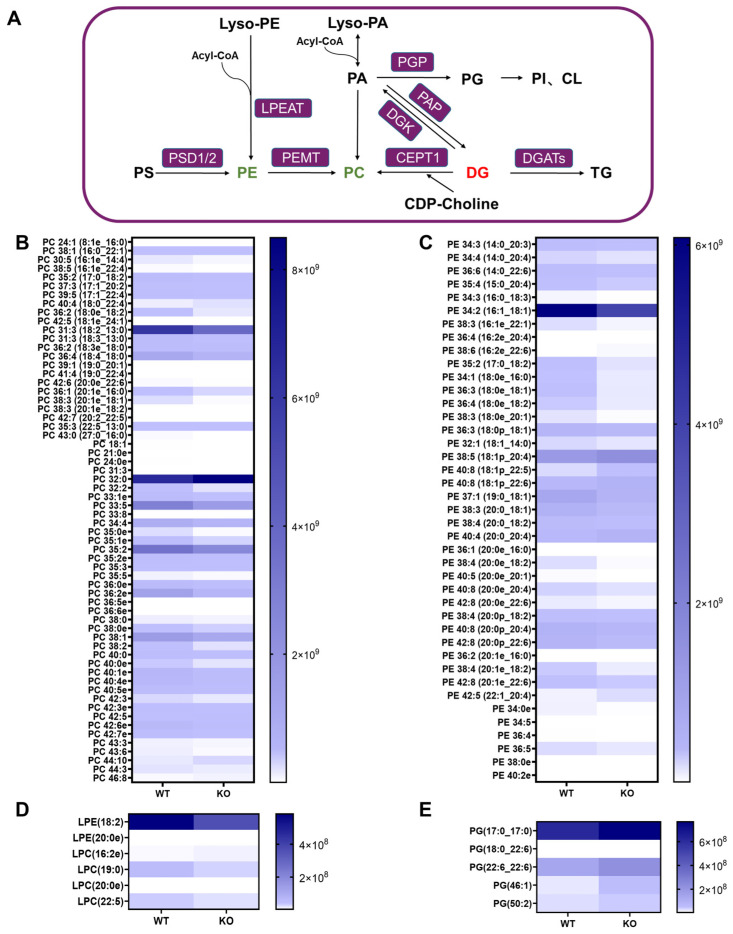
Altered phospholipid metabolism landscape in the *Hsd17b13* KO mice. (**A**) An illustrative diagram depicting various lipid metabolic pathways and their interconversion processes. A heatmap showing the differences in (**B**) PC, (**C**) PE, (**D**) LPE, LPC, and (**E**) PG levels between the WT and *Hsd17b13* KO mice. Lysophosphatidylethanolamine (Lyso-PE); acyl-coenzyme A (Acyl-CoA); lysophosphatidic acid (Lyso-PA); phosphatidic acid (PA); phosphatidylinositol (PI); cytidine diphosphate-choline (CDP-choline); phosphatidylserine decarboxylase 1/2 (PSD1/2); phosphatidylethanolamine N-methyltransferase (PEMT); lysophosphatidylethanolamine acyltransferase (LPEAT); choline/ethanolamine phosphotransferase 1 (CEPT1); phosphatidylglycerophosphate (PGP); phosphatidic acid phosphatase (PAP); diacylglycerol kinase (DGK).

By conducting a comprehensive lipidomic analysis, we identified significant perturbations in hepatic phospholipid metabolism in the *Hsd17b13* KO mice, implicating its important role in the pathogenesis of aging-related hepatitis. PCs are a class of 1,2-diacylglycerophospholipids and are essential components of cell membranes; they are also critical architectural lipids for membrane physical properties. The hepatic biosynthesis of PC occurs via multiple pathways, including those involving PE, phosphatidic acid (PA), and DG (Figure 4A). Under physiological conditions, ~70% of PC is synthesized via the CDP-choline pathway, while the remaining 30% originates from PE methylation mediated by PEMT [36]. A heatmap analysis of differentially expressed PC species revealed a systemic downregulation of hepatic PC pools in the *Hsd17b13* KO mice (Figure 4B). A total of 169 PC species showed changes in the KO mice, with 34 species being upregulated and 135 PC species being downregulated. Furthermore, PC O-18:2_13:0, PC O-18:4_18:0, PC O-33:5, and PC O-35:2 exhibited pronounced reductions in the *Hsd17b13* KO mice versus the WT controls. Meanwhile, we noticed that PC O-32:0 increased in the liver of the *Hsd17b13* KO mice.

Analogous to PC, PE serves as a critical structural and functional phospholipid in cellular membranes, orchestrating membrane integrity maintenance, fusogenic activity, and the dynamic regulation of membrane fluidity. Hepatic PE biosynthesis occurs via two distinct pathways: (1) the Kennedy pathway in the endoplasmic reticulum and (2) the PS decarboxylation pathway, a mitochondrial process converting PS to PE through decarboxylase-mediated catalysis [22]. Comparative lipidomic profiling revealed marked alterations in the abundance of PE species between the WT and *Hsd17b13* KO mice (Figure 4C). For instance, ether-linked phosphatidylethanolamines (alkyl-PEs, e.g., PE O-20:0_18:2 (FC = 0.26) and PE O-20:0_20:1 (FC = 0.26)) and diacyl-PE species (e.g., PE 14:0_22:6 (FC = 0.60) and PE 16:1_18:1 (FC = 0.65)) were coordinately downregulated in the liver of the *Hsd17b13* KO mice. In the *Hsd17b13* KO mice, the LPE 18:2, LPC 19:0, and LPC 22:5 levels were reduced, while the PG 46:1 and PG 17:0_17:0 levels were increased (Figure 4D,E).

### 3.5. Cer Metabolic Remodeling in the Hsd17b13 KO Mice

**Figure 5:**
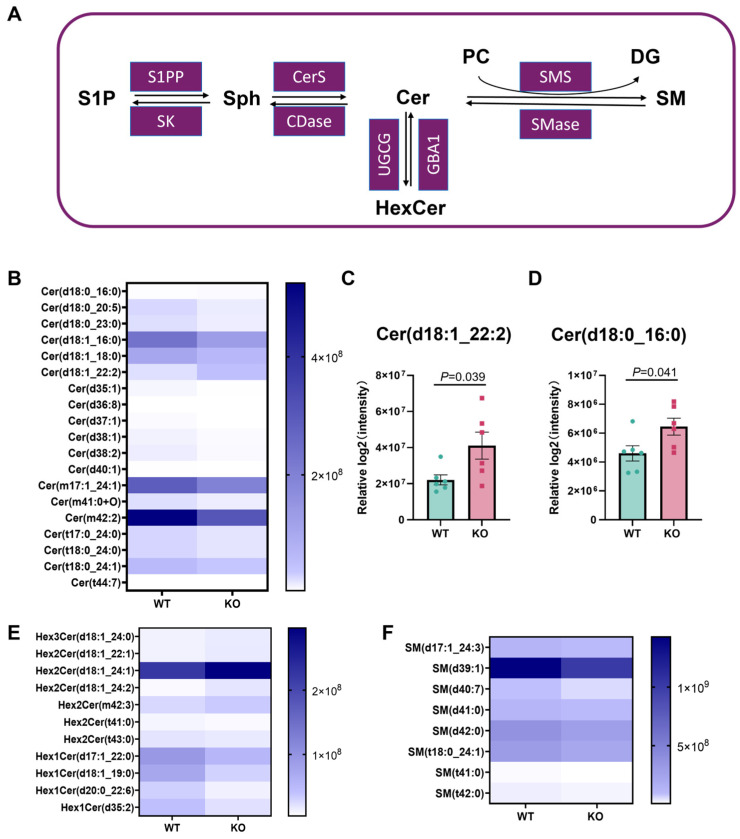
Cer levels are altered in the *Hsd17b13* KO mice. (**A**) Comprehensive schematic of Cer biosynthesis and metabolic crosstalk with the sphingolipid pathways. (**B**) Heatmap illustrating the significantly altered levels of Cer between the WT and *Hsd17b13* KO mice. (**C**,**D**) Comparative analysis of hepatic tissue levels of Cer (d18:1_22:2) and Cer (d18:0_16:0) between the WT and *Hsd17b13* KO mice. (**E**,**F**) Heatmap illustrating the significantly altered levels of hexosylceramide (HexCer) and SM between the WT and *Hsd17b13* KO mice (*n* = 6). Sphingosine-1-phosphate (S1P); sphingosine (Sph); sphingomyelin (SM); sphingosine-1-phosphate phosphatase (S1PP); sphingosine kinase (SK); ceramide synthase (CerS); ceramidase (CDase); UDP-glucose ceramide glucosyltransferase (UGCG); glucocerebrosidase 1 (GBA1); sphingomyelin synthase (SMS); sphingomyelinase (SMase).

Cer consists of a highly conserved sphingoid base backbone (typically d18:1 or d18:0) and a fatty acyl chain that varies in length. Specific Cer is known to play a critical role in liver injury, insulin resistance, apoptosis, and inflammatory cascades [37,38,39]. Here, we illustrate the key metabolic pathways of sphingolipids, focusing on the interconversions among sphingosine-1-phosphate (S1P), sphingosine (Sph), and Cer. The diagram showcases the enzymes involved in these transformations, including sphingosine-1-phosphate phosphatase (S1PP), sphingosine kinase (SK), ceramide synthase (CerS), ceramidase (CDase), UDP-glucose ceramide glucosyltransferase (UGCG), glucocerebrosidase 1 (GBA1), sphingomyelin synthase (SMS), and sphingomyelinase (SMase). Additionally, it depicts the synthesis and degradation processes of hexosylceramide (HexCer) and sphingomyelin (SM), providing a comprehensive view of the sphingolipid metabolic network (Figure 5A). Consequently, we measured the Cer levels in the two groups. Compared with the WT mice, the *Hsd17b13* KO mice had significantly increased levels of Cer (d18:0_16:0) (FC = 1.4) and Cer (d18:1_22:2) (FC = 1.86) (Figure 5B,D). As depicted in Figure 5A, the enzymes UGCG and GBA1 play crucial roles in the synthesis and degradation of HexCer from Cer. Meanwhile, some Hex1Cer lipids, such as Hex1Cer (d20:0_22:6) (FC = 0.4) and Hex1Cer (d35:2) (FC = 0.51), were decreased, while Hex2Cer (d18:1_24:2) and CerG3GNAc1 (d42:2) were increased (Figure 5E). As depicted in Figure 5A, the metabolic pathway involves the synthesis of sphingomyelin (SM) from Cer and PC, catalyzed by SMS, as well as its subsequent degradation back to Cer and DG by the action of SMase. The levels of sphingomyelin species, such as SM (d39:1) and SM (d40:7), in the *Hsd17b13* KO mice are shown in Figure 5F. The remodeling of Cer might contribute to the aging-related liver inflammation in the *Hsd17b13* KO mice.

### 3.6. Advanced Lipidomic Analysis of the Aged Hsd17b13 KO Mice

**Figure 6:**
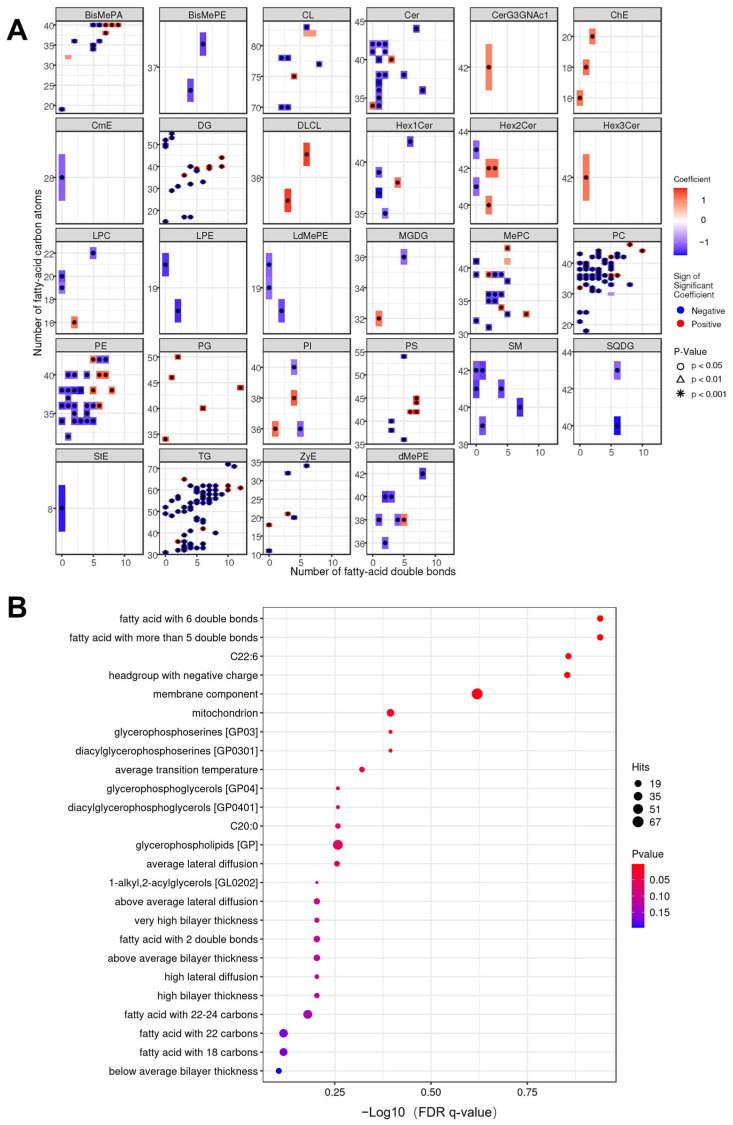
Advanced lipidomic analysis of the aged *Hsd17b13* KO mice. (**A**) Heatmap of the statistical differences in lipid structural characteristics. In the heatmap, rectangles represent different lipid structures of this category. The color indicates the level of the signal value, with red representing a high response and blue representing a low response. The *X*-axis represents the level of carbon saturation (the number of double bonds), and the *Y*-axis represents the number of carbon atoms in the lipids. (**B**) Pathway analysis bubble chart highlighting mitochondrial dysfunction in the aged *Hsd17b13* KO mice. The horizontal axis represents the –log10 value of the enrichment FDR q-value; the vertical axis indicates the names of the structures, functions, etc., in the LION database that are enriched. The color denotes the significance of the enrichment *p*-value, with redder colors indicating more significant enrichment results. The size of the bubble points represents the number of differentially enriched lipids, with larger sizes indicating a greater number of enriched lipids.

A systematic lipidomic topology analysis using the lipidomoR framework revealed profound age-dependent lipid remodeling in the livers of the *Hsd17b13* KO mice, characterized by the selective enrichment of very-long-chain (≥C24) polyunsaturated fatty acids (Figure 6A). In a lipidomic study of aging mice, elevated levels of polyunsaturated fatty acids were also reported in multiple tissues, including the liver, lung, muscle, bone marrow, and small intestine [39]. In contrast to the WT group, the *Hsd17b13* KO group showed the enrichment of multiple crucial pathways associated with lipid species that were differentially expressed. In particular, the changes in “fatty acid with 6 double bonds” and “fatty acid with more than 5 double bonds” were the most significant. Among these fatty acids, docosahexaenoic acid (22:6) was the most abundant FA with six double bonds (Figure 6B). Concurrently, there was also a significant enrichment of several lipids associated with mitochondria, indicating that the dysregulated lipid metabolism observed in our study has a crucial impact on the structure and function of mitochondria.

## 4. Discussion

MAFLD is the most prevalent chronic liver disease worldwide, yet effective pharmacological treatments remain limited. Growing evidence implicates HSD17B13 in the pathogenesis of multiple liver diseases. However, the conclusions drawn from these studies remain a subject of debate and controversy within the scientific community [12,32,40]. Our study revealed a notable increase in the average size of LDs accompanied by a reduction in LD number in the liver of *Hsd17b13* KO mice. Interestingly, these morphological changes were not associated with a substantial accumulation of hepatic neutral lipids (TG and TC). It is well-documented that TG is primarily synthesized by DGAT through acyl-CoA-dependent pathway. However, acyl-CoA-independent pathways may also be involved, including in the formation of TG from two molecules of DG via DGTA or the generation of TG from DG and MG under the action of PNPLA3. Our findings are inconsistent with those reported by Marion et al., who observed an increased accumulation of lipids in the liver of male *Hsd17b13* KO mice [32]. These discrepancies may be attributed to differences in the *Hsd17b13* gene targeting strategy and the age of the mice used in the studies. Their *Hsd17b13* KO mice were generated by replacing exons 1 and 2 of the *Hsd17b13* gene with a lacZ-expressing cassette through homologous recombination in embryonic stem cells, and the mice were 9 months old. However, our *Hsd17b13* KO mice were generated by introducing a deletion of four base pairs of the first exon of the *Hsd17b13* gene, and the mice were 22 months old.

LDs are dynamic organelles central to lipid and energy homeostasis and participate in numerous biological processes and activities [41]. Structurally, LDs comprise a neutral lipid core (primarily TGs, ChEs, and retinyl esters) surrounded by a phospholipid monolayer membrane studded with LD-associated proteins [42]. Over 200 structural and functional proteins, for example PLINs, play crucial roles in regulating lipid droplet homeostasis and mediating their interactions with organelles such as the endoplasmic reticulum (ER) and mitochondria [43]. Among these, HSD17B13 is one major hepatic-specific LD protein that play a critical role in lipid metabolism. The surface composition of LDs determines many of their properties, such as size, subcellular distribution, and interactions with other organelles [44]. Thus, the increase in the LD size and number observed in the aged KO mice may be associated with the function of HSD17B13, although the precise mechanisms remain to be elucidated. Further investigation is warranted to uncover the underlying mechanisms.

Notably, the LD monolayer membrane is enriched in PC, which constitutes ~50% of its phospholipid content, followed by other phospholipids, such as PI. The composition of the monolayer is similar to that of the bilayer of the ER [42]. In the aged *Hsd17b13* KO mice, concurrent disruptions in PC metabolism raise the possibility that LD enlargement caused by altered PC homeostasis. PC is the key building block of membrane bilayers and plays a major role in membrane-mediated cell signaling [45]. Its depletion has been linked to hepatic injury exacerbation under diabetic conditions, where PC supplementation mitigates cytotoxicity, oxidative stress, and inflammation [46]. A recent study reported that HSD17B13-mediated liquid-liquid phase separation drove hepatic inflammatory responses by enhancing platelet-activating factor-dependent leukocyte adhesion [47]. Here, we observed that HSD17B13 deficiency also caused enhanced inflammation in the aged mice. The heightened inflammatory infiltration observed in our *Hsd17b13* KO mice may be related to the reduction in PC levels. Whether PC depletion directly contributes to inflammation in this context warrant further investigation.

Regarding inflammation, it has been documented in the literature that a high-fat diet leads to the accumulation of DG and Cer, activates protein kinase C, and induces inflammatory pathways [48]. Our lipidomic analysis revealed significant upregulation of specific lipids, including DG 22:3_22:6, DG 40:4, DG 22:4_22:5, Cer (d18:0_16:0), and Cer (d18:1_22:2), in the aged *Hsd17b13* KO mice. These findings implicate these lipids as potential mediators of hepatic inflammation in HSD17B13 deficiency mice.

DG and PC share a profound and intricate relationship within the realm of lipid metabolism. PC biosynthesis primarily occurs via the Kennedy pathway, where CEPT1 catalyzes the conversion of CDP-choline and DG to PC in the endoplasmic reticulum. Similarly, in the Golgi apparatus, this conversion is catalyzed by choline phosphotransferase 1 (CHPT1) [49]. CEPT1 is a bifunctional enzyme that also catalyzes the synthesis of PE using CDP-ethanolamine as a substrate [50]. Specifically, PC can also be generated from PE by a finely regulated enzymatic process catalyzed by PEMT via three sequential methylations [51]. The dysregulation of their metabolism, particularly through PEMT activity and aryl hydrocarbon receptor signaling, is associated with the development and progression of MAFLD and MASH [52]. In our results, we observed a widespread reduction in PC and PE and an increase in DG in the aged *Hsd17b13* KO mice. This aligns with reports that HSD17B13 modulates CDP-choline levels to regulate PC biosynthesis [47]. The reduction in PC levels is likely related to the HSD17B13 deficiency, which also suggests the possibility of reduced expression or impaired enzymatic activity of CEPT1 or PEMT. 

Our study has some limitations. First, we focused exclusively on 22-month-old mice. It is necessary to study the liver lipid profiles of mice of different ages. This would allow us to demonstrate the effects of HSD17B13 deficiency across various age groups, and it would also enable us to detect changes in the lipid profiles of WT mice at different ages. Second, the absence of plasma lipidomic data represents a significant gap, as hepatic and circulating lipids are dynamically interrelated. Inclusion plasma lipidomic analysis data could have provided a more comprehensive understanding of HSD17B13 deficiency in systemic lipid metabolism. While we identified significant changes in specific lipid classes, our current understanding of the functions of lipids with diverse side chains remains rather limited. Therefore, more in-depth investigations into the functions of lipids are urgently required.

## 5. Conclusions

In conclusion, our comprehensive untargeted lipidomic analysis revealed substantial changes in the liver lipid profiles of aged mice with *Hsd17b13* gene knockout. Specifically, we observed significant alterations in the levels of TG, DG, PC, PE, PG, and Cer. These findings underscore the critical role of HSD17B13 in modulating neutral lipid and phospholipid metabolism in the livers of aging mice. Our results highlight HSD17B13 as a key determinant in maintaining hepatic lipid metabolic balance during the aging process, suggesting its potential as a therapeutic target for age-related metabolic dysregulation.

## Data Availability

All other data that support the findings of this study are available from the corresponding author upon reasonable request.
